# Supplementary figures and images for: Asthma severity as a contributing factor to cancer incidence: A cohort study
Source: PLoS One. 2021 May 13;16(5):e0250430. doi: 10.1371/journal.pone.0250430 (PMC8118340; doi:10.1371/journal.pone.0250430)

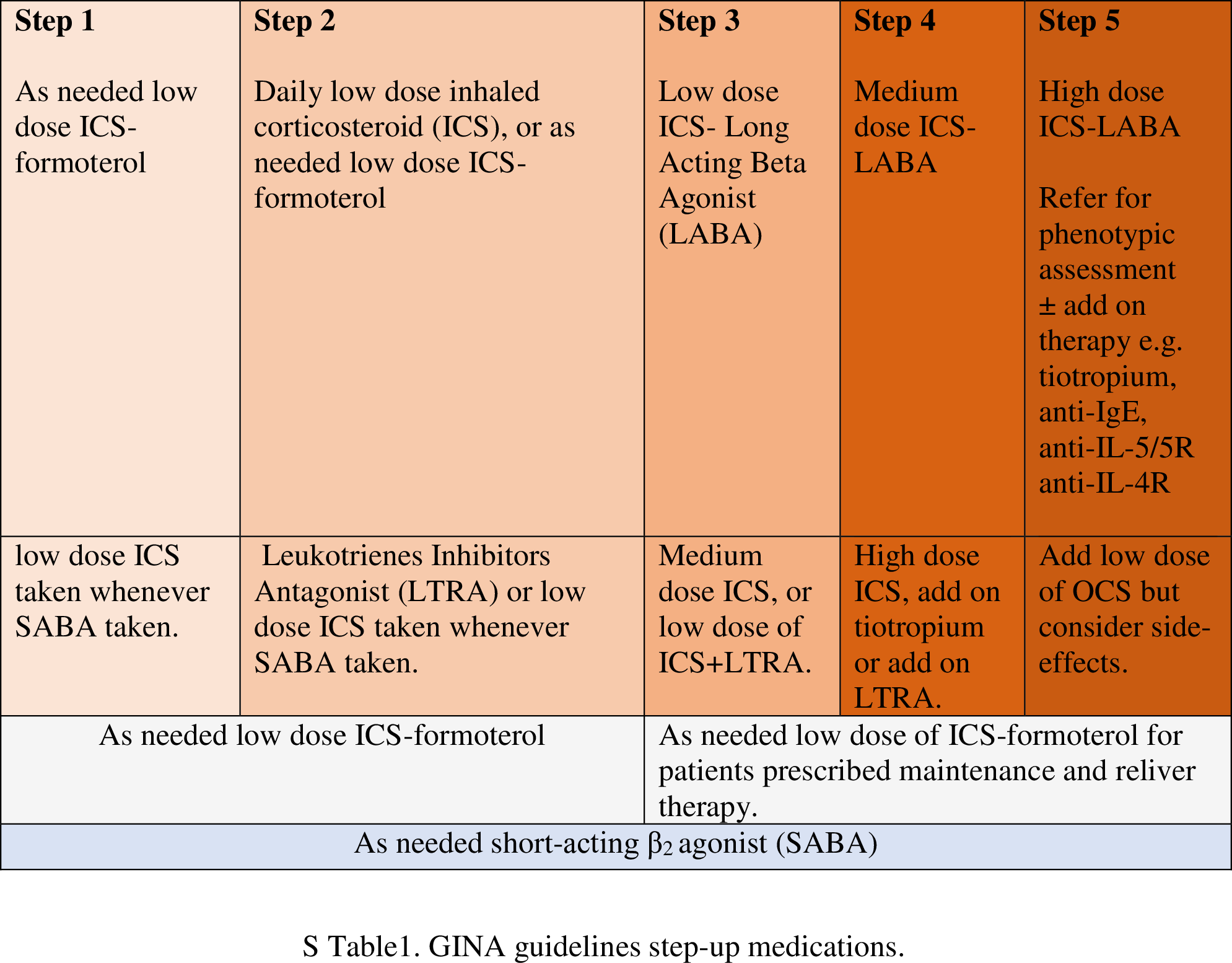

Supplement: S1 Table — (TIF) [file pone.0250430.s001.tif]

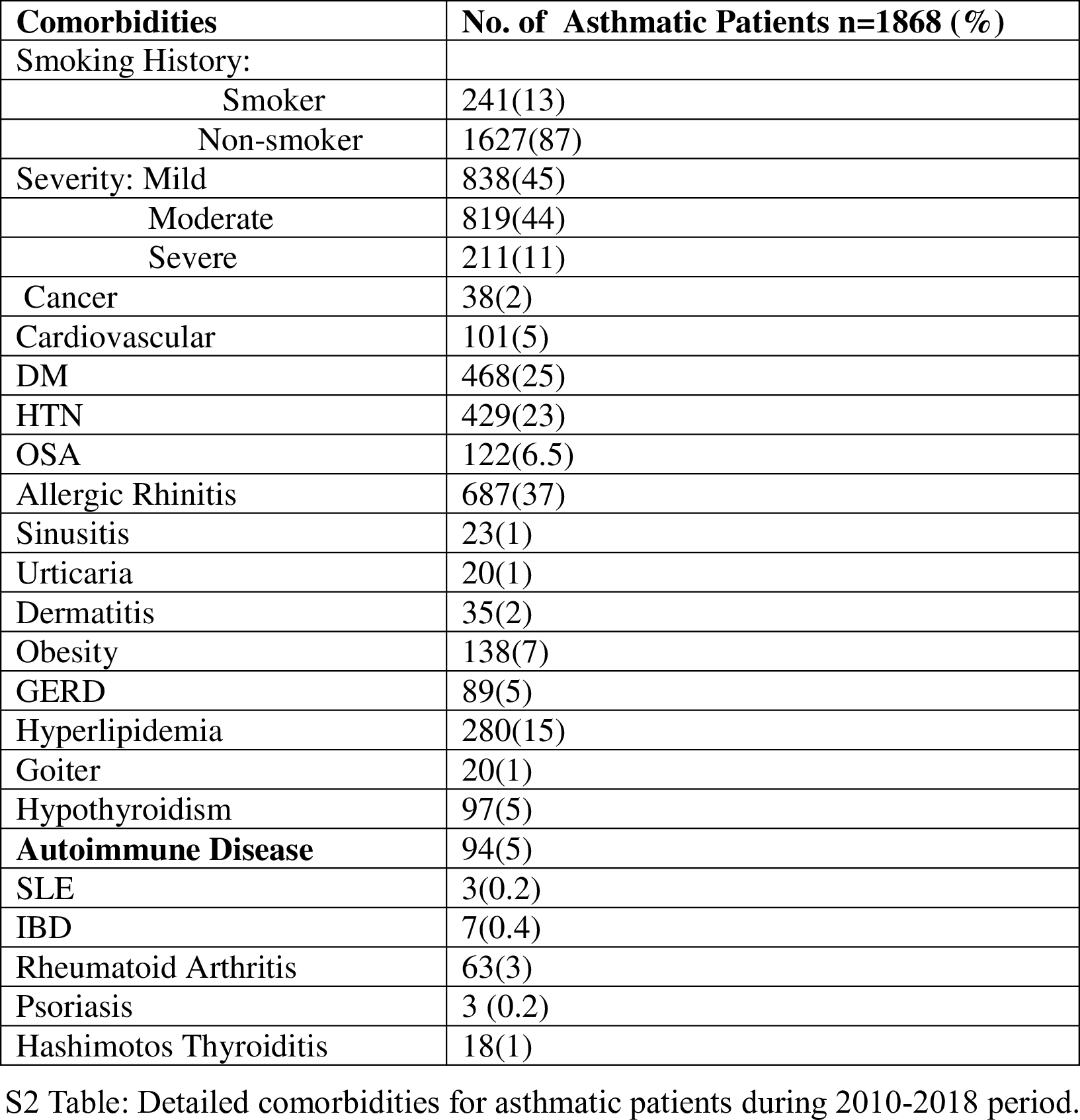

Supplement: S2 Table — (TIF) [file pone.0250430.s002.tif]

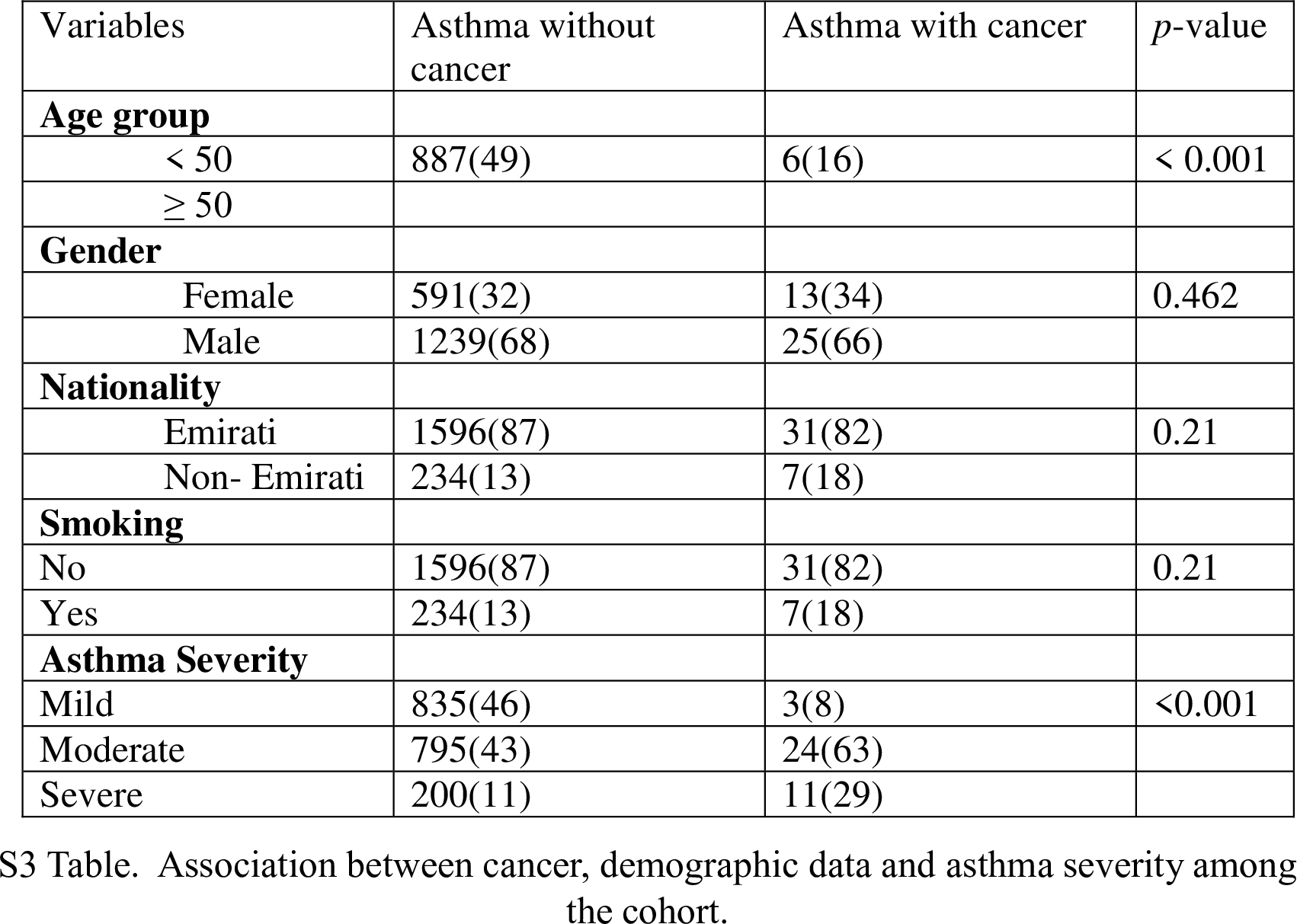

Supplement: S3 Table — (TIF) [file pone.0250430.s003.tif]

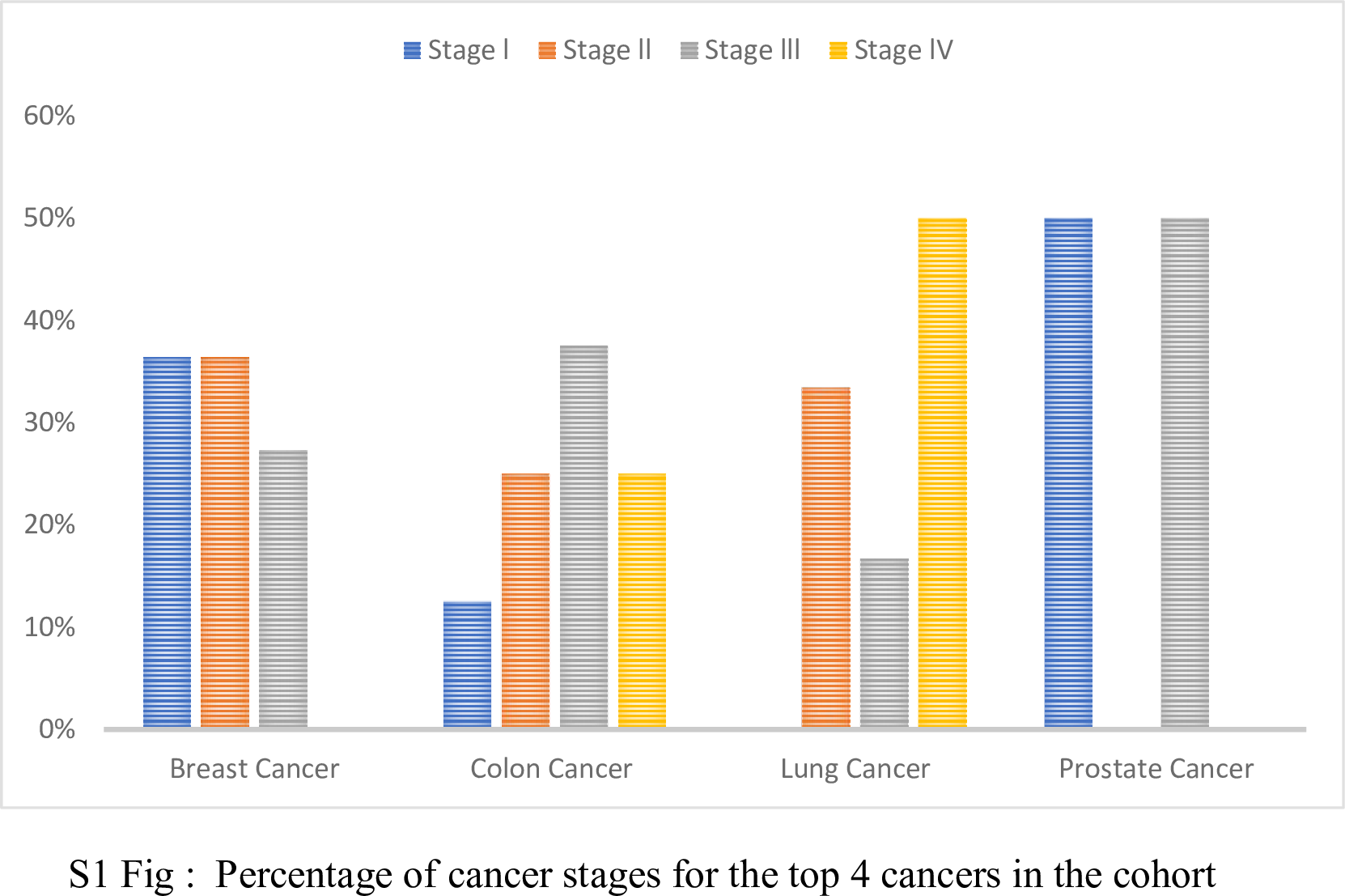

Supplement: S1 Fig — (TIF) [file pone.0250430.s004.tif]

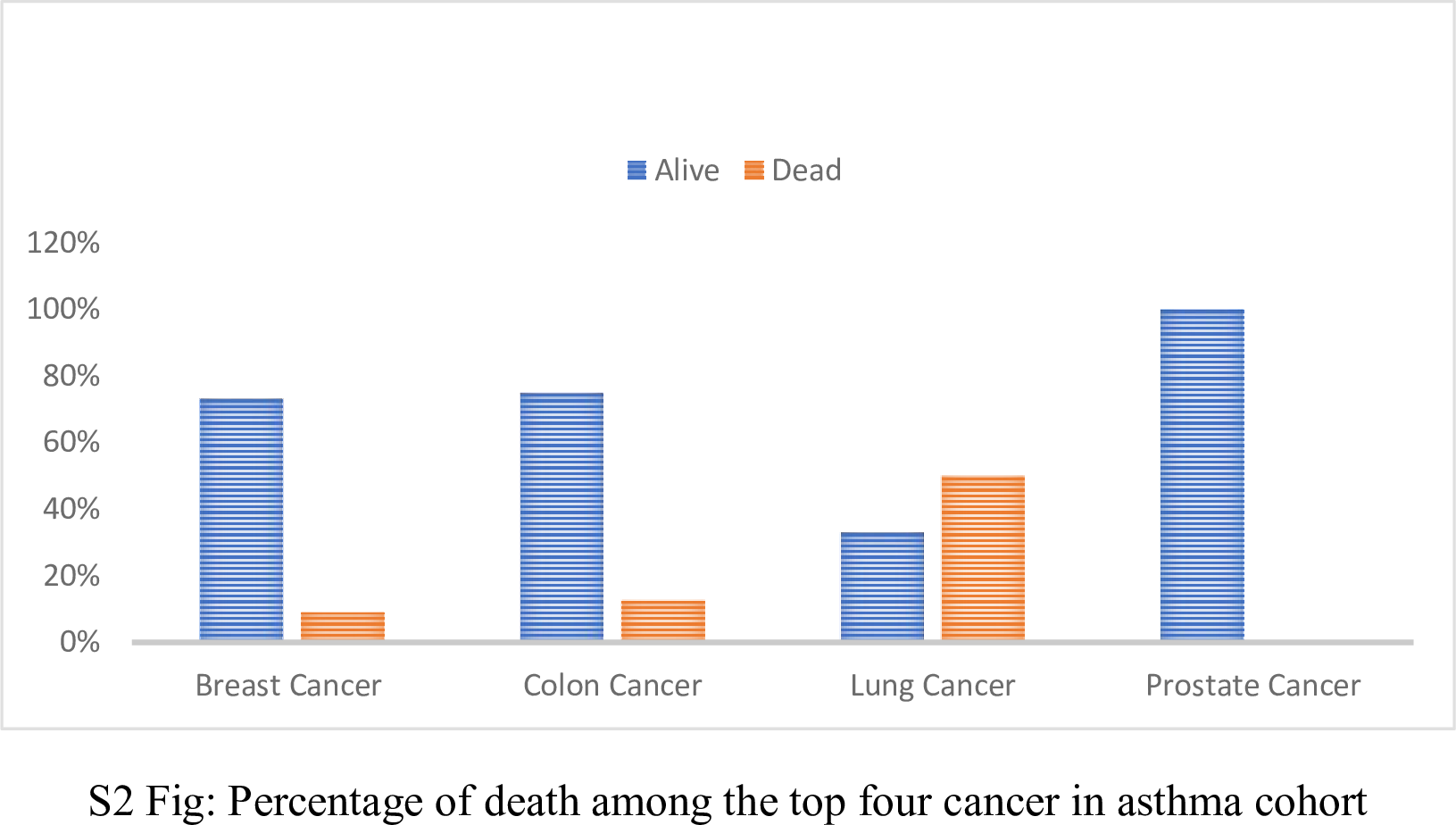

Supplement: S2 Fig — (TIF) [file pone.0250430.s005.tif]
