# Supplementary material for: Asthma severity as a contributing factor to cancer incidence: A cohort study
Source: PLoS One. 2021 May 13;16(5):e0250430. doi: 10.1371/journal.pone.0250430 (PMC8118340; doi:10.1371/journal.pone.0250430)
Supplement: Attachment — Submitted filename: Response to reviewers Final.docx [file pone.0250430.s008.docx]

Journal Requirements:

When submitting your revision, we need you to address these additional requirements.

1. Please ensure that your manuscript meets PLOS ONE's style requirements, including those for file naming. The PLOS ONE style templates can be found at

https://journals.plos.org/plosone/s/file?id=wjVg/PLOSOne_formatting_sample_main_body.pdf and

[https://journals.plos.org/plosone/s/file?id=ba62/PLOSOne_formatting_sample_title_authors_affiliations.pdf](about:blank)

2. Please review your reference list to ensure that it is complete and correct. If you have cited papers that have been retracted, please include the rationale for doing so in the manuscript text, or remove these references and replace them with relevant current references. Any changes to the reference list should be mentioned in the rebuttal letter that accompanies your revised manuscript. If you need to cite a retracted article, indicate the article’s retracted status in the References list and also include a citation and full reference for the retraction notice.

3. In the ethics statement in the manuscript and in the online submission form, please provide additional information about the patient records/samples used in your retrospective study, including the date range (month and year) during which patients' medical records/samples were accessed.

If patients provided informed written consent to have data from their medical records used in research, please include this information.

We thank the editor for pointing this out. We have added the following paragraph in lines 85-89.

“All patient records were collected from the computerized information health system in Dubai Health Authority. All records in the outpatient and inpatient register, containing a hospital-discharge diagnosis of asthma. The data accessed were from 24/9/2019 – 30/4/2020. All patients were consented. Patient records were fully anonymized prior to access.”

4. Please include a copy of Table 6 which you refer to in your text on page 11.

We thank the editor for pointing out the anomaly. In fact, there is no Table 6. Table 4 was mislabeled as Table 6. This correction has been made in line 224.

5. We note you have included a table to which you do not refer in the text of your manuscript. Please ensure that you refer to Table 4 in your text; if accepted, production will need this reference to link the reader to the Table.

Thank you for pointing out the anomaly. In fact, there is no Table 6. Table 4 was mislabeled at Table 6. This correction has been made in line 224.

[Note: HTML markup is below. Please do not edit.]

Reviewers' comments:

Reviewer's Responses to Questions

**Comments to the Author**

1. Is the manuscript technically sound, and do the data support the conclusions?

The manuscript must describe a technically sound piece of scientific research with data that supports the conclusions. Experiments must have been conducted rigorously, with appropriate controls, replication, and sample sizes. The conclusions must be drawn appropriately based on the data presented.

Reviewer #1: Yes

Reviewer #2: Yes

2. Has the statistical analysis been performed appropriately and rigorously?

Reviewer #1: Yes

Reviewer #2: Yes

3. Have the authors made all data underlying the findings in their manuscript fully available?

The [PLOS Data policy](http://www.plosone.org/static/policies.action#sharing) requires authors to make all data underlying the findings described in their manuscript fully available without restriction, with rare exception (please refer to the Data Availability Statement in the manuscript PDF file). The data should be provided as part of the manuscript or its supporting information, or deposited to a public repository. For example, in addition to summary statistics, the data points behind means, medians and variance measures should be available. If there are restrictions on publicly sharing data—e.g. participant privacy or use of data from a third party—those must be specified.

Reviewer #1: Yes

Reviewer #2: Yes

4. Is the manuscript presented in an intelligible fashion and written in standard English?

PLOS ONE does not copyedit accepted manuscripts, so the language in submitted articles must be clear, correct, and unambiguous. Any typographical or grammatical errors should be corrected at revision, so please note any specific errors here.

Reviewer #1: Yes

Reviewer #2: Yes

5. Review Comments to the Author

Please use the space provided to explain your answers to the questions above. You may also include additional comments for the author, including concerns about dual publication, research ethics, or publication ethics. (Please upload your review as an attachment if it exceeds 20,000 characters)

Reviewer #1: This retrospective single-center cohort study evaluates the incidence of all cancer types in a population of asthma patients compared to a control population followed over a 9-year time interval. The role of asthma as a potential cancer risk factor is then evaluated.
This cohort study is well constructed. I would suggest a re-reading of the work in order to carry out a grammatical revision; for example on line 234: "the shows that ..."

We thank the reviewer for taking the time to read through the manuscript. We have carefully gone through the manuscript and corrected the grammatical and other linguistic errors identified.

The introduction investigates the relationship between cancer and chronic inflammatory diseases focusing on asthma. In the introduction we find: "Recent studies demonstrated that the association between history of allergic disorders and asthma, and the risk of cancer varied by cancer types". I would ask the authors to develop this part of the introduction more.

We thank the reviewer for raising this point and agree that it is important to expand on it to introduce the fact that the relationship between the association of asthma and cancer. We have added the following statement in line 61-64:

“A protective effect of atopic diseases against pancreatic cancer has been shown consistently in case-control studies but not in cohort studies. Most studies on atopic diseases and non-Hodgkin lymphoma or colorectal cancer reported an inverse association.”

Moreover, this completes the statement in line 64-65:

“Inversion association between cancer and asthma have been reported in case-control studies of glioma [22, 23], and leukemia [24, 25].”

Reviewer #2: This paper is very well written. The results are very interesting for readers.The authors' conclusions are consistent with the data reported in the paper. I n conclusion the paper deserves to be published.

We thank the reviewer for their complimentary comments and for giving their time to go through the manuscript. We hope that the manuscript can add to the existing body of knowledge on the association between asthma and lung cancer.

6. PLOS authors have the option to publish the peer review history of their article ([what does this mean?](https://journals.plos.org/plosone/s/editorial-and-peer-review-process#loc-peer-review-history)). If published, this will include your full peer review and any attached files.


If you choose “no”, your identity will remain anonymous but your review may still be made public.


**Do you want your identity to be public for this peer review?** For information about this choice, including consent withdrawal, please see our [Privacy Policy](https://www.plos.org/privacy-policy).

Reviewer #1: No

Reviewer #2: **Yes:** Urso Domenico Lorenzo
